# Immunogenic Effects and Clinical Applications of Electroporation-Based Treatments

**DOI:** 10.3390/vaccines12010042

**Published:** 2023-12-30

**Authors:** Mariangela De Robertis, Emanuela Signori

**Affiliations:** 1 Department of Biosciences, Biotechnology and Environment, University of Bari ‘A. Moro’, 70125 Bari, Italy; 2 Laboratory of Molecular Pathology and Experimental Oncology, Institute of Translational Pharmacology, CNR, 00133 Rome, Italy

Immunotherapy can now be regarded as an attractive approach for cancer and infectious disease treatments. A variety of novel and varied therapeutic approaches are being discovered through immunology research. Among them, some treatment strategies are based on electrotransfer, also called electroporation (EP). EP is an efficient and safe method that uses voltage pulses to make temporary openings in the membrane, through which drugs or genes can be delivered to the target cells by electrotransfer [1]. As evidenced by past and present studies, EP impacts the immune system response by recruiting different types of immune cells and affecting both local and systemic immune reactions [2].

Because of this property, many studies of immunization by electroporation have been conducted in both the clinical [3] and veterinary [4] fields. EP has indeed emerged as a widely accepted technological platform for Gene Electrotransfer (GET) and Electrochemotherapy (ECT), which are two Reversible Electroporation (RE) approaches, but also for Irreversible Electroporation (IRE) [5]. These therapeutic options are all based on the administration of electric pulses in different conditions and for different purposes. They are employed to destroy cancerous cells with thermal heat (IRE) [6]; for the administration of genes coding immunotherapeutic molecules and/or antigens (GET) [7]; and for the intra-tumoral delivery of drugs such as Bleomycin and Cisplatin (ECT) [8]. All these procedures have demonstrated their capability to recruit different immune cells for innate and adaptive immune responses. However, many efforts are underway in in vitro and in vivo studies to optimize EP protocols to minimize tissue damage and improve gene transfection efficiency [9,10,11,12] or drug administration [13,14].

This Special Issue reflects the variety in studies that focus on EP as an effective and secure method to perform IRE treatments or to deliver drugs or genes into the target cells. These articles emphasize the main immunological outcomes and improvements of the immune system response elicited by genetic vaccines and/or immunomodulatory molecules administered by themselves or together with other therapeutic treatments by EP. It includes two articles and two reviews which address topics related to this subject.

Gong et al. reviewed the most recent advances in EP-related therapies and the synergy with immunotherapy in cancer treatment. Both Reversible and Irreversible Electroporation-based approaches (RE and IRE) are considered, depending on the electric pulse parameters and the electrophysiology of the target cell. As the authors state, RE transfers functional genes or drugs to target cells, resulting in cell death by several pathways, such as apoptosis, mitotic catastrophe, or pseudoapoptosis, while IRE is a technology that directly ablates a lot of tissue without harmful thermal effects. Using RE and IRE, it is also possible to activate an immune response that targets tumors systemically and enhances the results of immunotherapy. Further, recent progress related to the application of EP and the synergistic effects of EP-related therapies and immunotherapy applied to cancer treatment is summarized.

Luz et al. reviewed the latest clinical outcomes and immune system impacts of RE-based therapies. Specifically, they outlined the physical changes in the cell membrane, the specificities of bleomycin administration, and the immune effects of both ECT and GET, two important EP-based therapies for cancer in humans and animals.

Polajže et al. explored how the cell death that triggers the immune system in EP-based therapies is influenced by the characteristics of the pulse waveform. Traditionally, different but typical pulse lengths of 100 microseconds and 1–50 milliseconds were used to perform EP-based therapies such as ECT, GET, and IRE. However, in vitro studies have shown that almost any pulse length (millisecond, microsecond, nanosecond) and pulse type (monopolar, bipolar-HFIRE) can be used to administer these treatments, leading to varying efficacy. Therefore, the authors examined if different pulse lengths and pulse types induce different or similar activations of the immune system by measuring the damage-associated molecular pattern (DAMP) release. Indeed, in EP-based therapies, the activation of the immune response can influence treatment outcomes, and the ability to control and predict immune responses could enhance the treatment. The study indicates that DAMP release can vary when different pulse lengths and pulse types are applied. The most immunogenic seem to be nanosecond pulses, as they can induce the release of all three main molecules that indicate cell damage (ATP, HMGB1, and calreticulin). Conversely, the least immunogenic seem to be millisecond pulses. In this case, only ATP release was seen, and this probably occurred due to the increased porosity of the cell membrane. Overall, the study concludes that DAMP release and immune response in EP-based therapies can be regulated though pulse length.

D’Alessio et al. investigated the immunological response elicited by an increasingly nicked plasmid DNA vaccine delivered using EP. This study considered the fact that DNA molecules are thought to be more stable than mRNA, which is why the latter requires a controlled cold chain to be effective. Accordingly, the authors explored how temperature and molecular isoforms associated with different levels of DNA integrity impact the immune effect of plasmid DNA vaccines delivered via EP. As a model, they used COVID-eVax, which is a DNA vaccine against the SARS-CoV-2 spike protein’s receptor binding domain (RBD), and applied either an accelerated stability protocol or a lyophilization protocol to it. Unexpectedly, the authors demonstrated that the immune response induced in vivo was only mildly influenced by the percentage of open circular DNA. This suggests that plasmid DNA vaccines remain effective when stored at higher temperatures; this is an important property that could make them more easily used in low-/middle-income countries.

A variety of novel and different therapeutic approaches for cancer and infectious diseases are being discovered through immunology research. This Special Issue provides an interesting up-to-date overview of many aspects related to electroporation and its capability to influence the immune system response, pointing out advancements and perspectives for future treatments.
